# Adeno-associated virus infection and its impact in human health: an overview

**DOI:** 10.1186/s12985-022-01900-4

**Published:** 2022-10-31

**Authors:** Thaís B Sant’Anna, Natalia M Araujo

**Affiliations:** Laboratory of Molecular Virology, Oswaldo Cruz Institute, FIOCRUZ, Rio de Janeiro, Brazil

**Keywords:** Adeno-associated virus, Pathogenesis, Tumorigenesis, Hepatocellular carcinoma, Cervical cancer, Reproductive disorders, Wildtype AAV, Gene therapy, Human health

## Abstract

Discovered as a contaminant of adenovirus stocks in the 1960s, adeno-associated virus (AAV) is a mono-stranded DNA virus that depends on helper factors to replicate. Even though AAV is endemic in the human population (35–80%), it is remarkable that many issues concerning the natural infection by this virus remain unanswered. In this study, we reflect on the main basic aspects of AAV biology and provide an overview of the studies exploring the impact of AAV infection on human health, focusing on three major research areas including, (i) cervical and (ii) liver cancer, and (iii) reproductive system disorders. Conflicting results have been obtained into the association of AAV infection with the occurrence of adverse reproductive outcomes, such as placental complications, spontaneous abortion, and fertility disorders, or with a protective role in HPV-related cervical carcinogenesis. Noteworthy, recent reports have identified AAV insertional mutagenesis as a novel risk factor for the development of hepatocellular carcinoma. This latest finding raises concern regarding the widespread usage of AAV vectors in liver-targeted gene therapy.

## Introduction

### Adeno-associated virus

Adeno-associated virus (AAV) was discovered as a contaminant in a simian adenovirus type 15 preparation in 1965 [1, 2]. Since then, AAV has been successfully developed into therapeutic vector, with Glybera (alipogene tiparvovec) becoming the first gene therapy medication to be authorized [3]. AAV infection is asymptomatic and can last a lifetime. The lack of identifiable associated disease and the unique capacity of recombinant AAV (rAAV) vectors to transduce dividing and non-dividing cells with high efficiency, long-term transgene expression, low immunogenicity, and selective tissue tropism make AAV appealing for gene therapy [4].

Within the *Parvoviridae* family, AAV belongs to the *Dependoparvovirus* genus. There are at least 13 naturally occurring serotypes, each with a different tissue tropism [5]. AAV infects various animal species, including humans, and is found worldwide, with seroprevalence varying from roughly 35–80% in the human population, depending on the AAV serotype and cohort analyzed [6, 7].

AAV can only replicate in the presence of helper factors, which are provided by helper virus coinfections. Adenovirus type 5 (AdV5) and herpes simplex virus type 1 (HSV-1) are well-studied AAV helper viruses. Although less well studied, many other members of the herpesvirus family have been shown to support productive AAV replication, such as human cytomegalovirus (HCMV), herpes simplex virus type 2 (HSV-2), varicella zoster virus (VZV), and human herpesvirus 6 (HHV-6) (reviewed in [8]). Recently, human bocavirus 1 was demonstrated as a helper virus during AAV replication [9]. Interestingly, AAV replication can be triggered by treating AAV-infected cells with physical or chemical carcinogens, indicating that it is not intrinsically dependent on viral coinfections but rather on a significant alteration in the cellular environment [10, 11]. Without helper factors, AAV transfers its genome into the host cell, where most copies are eliminated within a short time, but other AAV genomes stay indefinitely. Long-term persistence is thought to occur mainly in an episomal, circular form. Latent AAV reactivates after coinfection with a helper virus, resulting in the emergence of progeny virus [8].

### AAV biology

The AAV virion is a non-enveloped icosahedral particle with a single-stranded DNA genome. The most thoroughly researched serotype of AAV is type 2 (AAV2), which serves as the AAV family’s prototype. Because most vector expertise has been gained with AAV2, we will utilize information about this serotype to explain generic AAV properties. The AAV2 virion has a diameter of around 20 nm and is made up of 60 copies of the three capsid proteins VP1, VP2, and VP3 in a 1:1:10 ratio. The VP1 and VP2 proteins share the VP3 sequence and contain extra residues at their N-termini. A conserved phospholipase A2 region found at the N-terminus of VP1 has been linked to viral escape from endosomes and is required for infectivity [12]. The VP2 protein is not required for infection or assembly [13]. The core of VP3 protein is formed by a conserved β-barrel motif consisting of antiparallel β-sheets. Other parvoviruses have this pattern, but the interstrand loops are variable, and they determine receptor usage and serology [14]. Understanding the molecular interactions of virus particles has relied heavily on structural and genetic data. X-ray crystallography and cryo-electron microscopy have been used to identify the structural images of numerous AAV capsids [14–17]. At the threefold axis, where proteins join together to create three clusters of peaks on the virion’s surface structure, there are several interactions between capsid subunits [14].

**Fig. 1:**
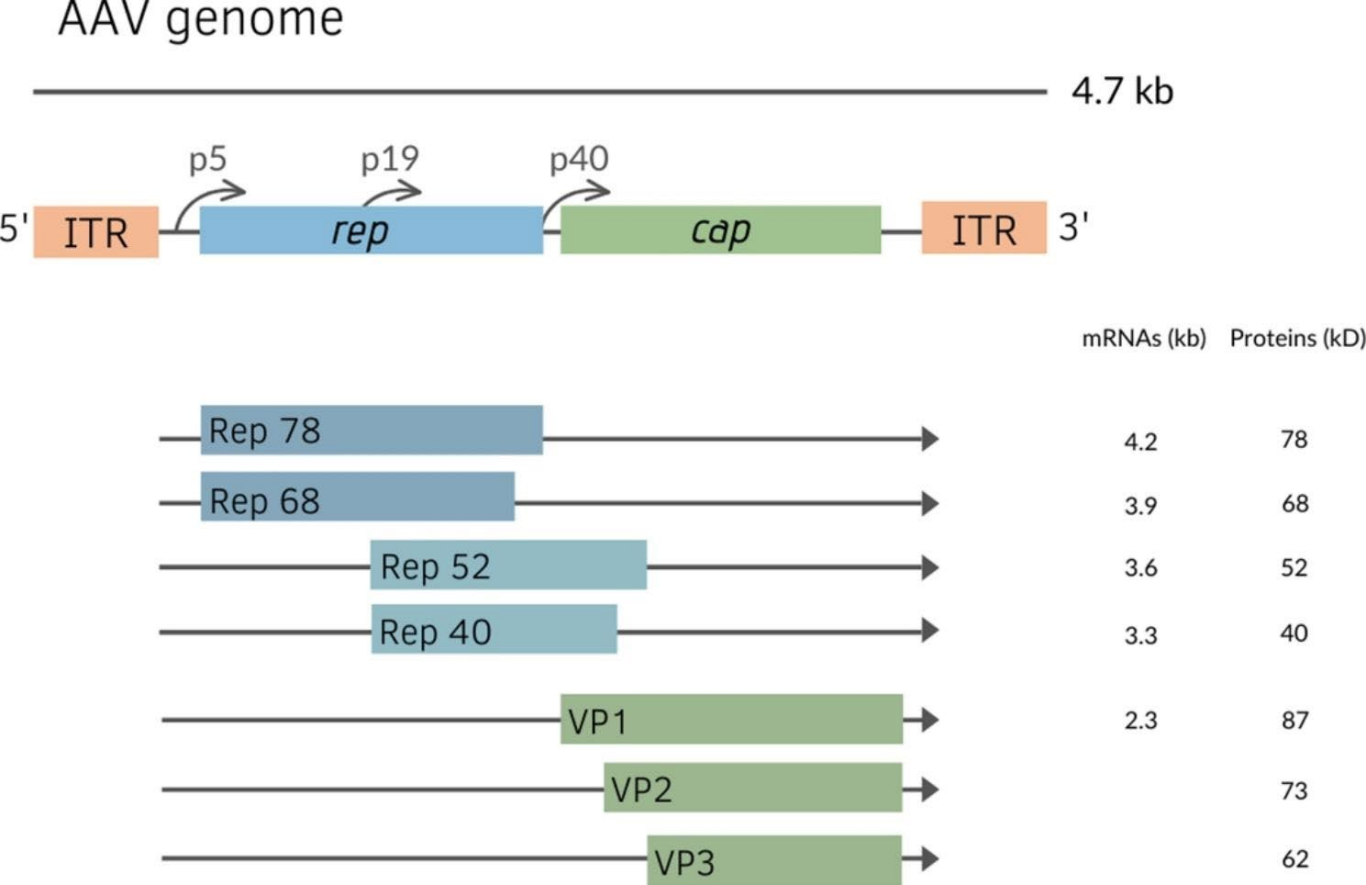
Transcriptional map of the AAV genome for major viral structural and nonstructural proteins. Spliced and unspliced forms of all three major transcripts are shown (for details, see text).

The AAV genome is a 4.7 kb molecule of single-stranded DNA. The positive and negative strands are packaged in separate premade particles with equal efficiency. Inverted terminal repeats (ITRs) create T-shaped, base-paired hairpin structures at each genome end and include cis-elements necessary for replication and packaging. Four nonstructural proteins necessary for replication (Rep78, Rep68, Rep52, and Rep40) and three structural proteins that make up the capsid (VP1, VP2, and VP3) are encoded by two genes (rep and cap). The p5, p19, and p40 viral promoters are recognized by their relative map location within the viral genome. Although different AAV serotypes have varied transcription patterns, all AAV2 transcripts have a single intron [18]. Rep78 and Rep52 are encoded by unspliced RNAs, whereas Rep68 and Rep40 are expressed by spliced RNAs (Fig. 1).

After binding to a primary receptor, AAV enters the cell via endocytosis [19]. It was discovered that various serotypes attach to distinct cell receptors. AAV2, AAV3, and AAV6 bind to heparan sulfate proteoglycan (HSPG), AAV1 to sialic acids, and AAV9 to N-linked galactose [19]. Coreceptors were proposed to have a role in AAV attachment and uptake, but further investigations failed to substantiate their importance [20–23]. Remarkably, AAV2 isolated directly from human tissues did not attach to HSPG, suggesting that lab strains have adapted to cell culture and that other AAV2 receptors exist in vivo [24]. A transmembrane protein termed AAV receptor (AAVR) was a necessary component for AAV transduction for many serotypes in a previous genetic screen [21]. In addition, GPR108 was another critical component for AAV entrance, which was suggested to function downstream in the same pathway as AAVR [25]. AAVR is found on the cell surface and is carried to the trans-Golgi network (TGN) in a retrograde endosomal way. Several endocytic routes have been proposed to play a role during the entrance. However, the clathrin-independent carrier (CLIC)/GPI-anchored protein-enriched early endosomal compartments (GEEC) pathway has been proven to be the most important endocytic route of infection [26, 27].

The precise method of AAV tracking from early endosomes to the cytoplasm is unknown. According to one scenario, AAV is carried from early endosomes to the TGN/Golgi apparatus, where it escapes and reaches the nucleus [28]. The transfer of AAV2 to the Golgi apparatus was revealed to be crucial for transduction, giving support to the hypothesis of retrograde endosomal transport. Retrograde transport via the endosomal system is a highly controlled and selective mechanism that allows the cell to recover and recycle proteins and lipids from the plasma membrane, allowing them to be localized to the Golgi (TGN and Golgi apparatus) and the endoplasmic reticulum (ER). Low endosomal pH and the action of proteases cause a conformational shift in the AAV capsid, exposing the N-terminal region of the major capsid protein, VP1 [29]. This so-called VP1 unique region (VP1u) comprises a phospholipase A2 domain (PLA2) and also a nuclear localization signal, enabling escape into the cytoplasm as well as nuclear import [12, 30–32]. Once within the nucleus, AAV2 was shown to accumulate in the nucleoli in an infectious form [33]. The process of AAV uncoating is poorly understood and appears to be a limiting step in AAV transduction [34, 35]. AAV may integrate into the host genome in the presence of Rep78, primarily at a locus on chromosome 19 designated AAVS1 [36, 37]. Although AAV genome integration is possible, multiple studies have demonstrated that integration inside AAVS1 and outside is rare, with just 0.1–0.5% of added infectious particles integrating [38, 39]. In cell culture and in vivo, the genomes of AAV-derived viral vectors were discovered to circularize over time, confirming the idea of a primarily circular episomal state during latency [40]. The binding of Rep78 and Rep68 to a particular region inside the p5 promoter, known as the Rep binding element (RBE), inhibits transcription during AAV latency, whereas the binding to the RBE sequence within the ITR stimulates transcription [41]. AAV enters its lytic stage after co-infection with a helper virus, which results in genome amplification and packaging.

During the construction of AAV-based vectors for gene therapy, a significant portion of the native AAV genome is deleted and the rep and cap genes are provided in trans. Briefly, rAAV vectors are commonly produced by triple plasmid transfection providing the AAV ITRs flanking a transgene cassette with a therapeutic gene of interest, the AAV rep and cap genes, and helper virus genes. The rAAV genomes are replicated and packaged into AAV capsids, which can be purified by different methods [42].

#### AAV infection in humans

Even though AAV has been studied for over 50 years, little is known about the virus’s natural infection. This fact may be even more surprising given that anti-AAV antibodies are found in up to 80% of the human population [6, 7]. AAV has been found in human blood cells, cervix uteri, penis, semen, liver, epithelial cell brushings, endometrium, amniotic fluid, and abortion material [43–46]. AAV may be transmitted through direct contact with an infected individual or indirect contact with the contaminated environment. Transmission routes include respiratory, gastrointestinal, and possibly sexual transmission. A concern for vertical transmission from mother to fetus also exists [46–48].

Very few studies addressing the impact of AAV infection on human health are available, and the results are conflicting. In this review, investigations on this subject were grouped into three major research topics (AAV infection and cervical cancer; AAV infection and reproductive system disorders; AAV infection and liver cancer), and were considered in the subsections below.

#### AAV infection and cervical cancer

With a reported 604,127 new cases and 341,831 deaths in 2020, cervical cancer is predicted to be the fourth leading cause of cancer and the third highest cause of cancer-associated mortality in women worldwide [49].

It has been proposed that AAV can be sexually transmitted, possibly in combination with herpes or papilloma viruses [54, 57]. High-risk human papillomavirus (HPV) infection plays a critical role in the natural history of cervical cancer [50]. During HPV infection, two viral oncogenic proteins, E6 and E7, play a crucial role in the development of cervical cancer by interacting with p53 and retinoblastoma protein (pRb) to render these cellular regulatory proteins inactive [51, 52]. AAV has been demonstrated to suppress HPV-induced cell transformation in vitro, which is mediated by the AAV Rep 78 protein [53–55]. Furthermore, the expression of H-*ras* and H-*fos* genes along with HPV oncogenes is inhibited by Rep 78 [56]. These collective studies suggest that AAV could be directly related to the suppression of HPV infection and may be thus associated with a reduced risk for HPV-related cervical neoplasia.

In line with the idea of a protective effect, Mayor et al. (1976) demonstrated a less likelihood of cervical cancer development in HPV-infected individuals in the presence of AAV since 85% of healthy women showed to be seropositive for AAV, while in cervical cancer patients AAV antibodies could only be detected in 14% of the cases [57]. Consistent with this observation, Georg-Fries et al. (1984) showed that sera from patients with cervical carcinoma revealed average titers of AAV antibodies well below those of age-matched control groups [58]. It was shown by Coker et al. (2001) that AAV positivity was significantly associated with a decreased risk of high-grade squamous intraepithelial lesion, suggesting that AAV may play a protective or inhibitory role in late-stage cervical carcinogenesis [59]. Furthermore, Agorastos et al. (2008) found that the prevalence of AAV was significantly lower in HPV-positive than HPV-negative patients (*P* = 0.0009, *P* = 0.00001, and *P* = 0.0225, for women with low-grade cervical lesions, high-grade cervical lesions, and cervical cancer, respectively). In contrast, no difference in the frequency of AAV DNA between HPV-positive and HPV-negative unaffected (control) women was observed [60]. More recently, Freitas et al. (2012) investigated the prevalence of AAV and HPV DNAs in cervical samples of HIV-seropositive and -seronegative women in Brazil. AAV-HPV co-infected women showed a lower rate of cervical intraepithelial neoplasia development compared with those infected only with HPV. HIV infection does not appear to influence AAV prevalence or AAV-HPV co-infection [61].

On the other hand, several studies did not support the protective role of AAV in cervical tumorigenesis. Strickler et al. (1999) found no relationship between AAV antibodies and the presence or grade of neoplasia in either Jamaican study subjects or women enrolled in a U.S. cervical cancer case [62]. In Odunsi et al. (2000), AAV DNA was not frequently present in either standard control cervical samples or cervical intraepithelial neoplasia, thus not supporting the hypothesis that AAV may be protective against cervical cancer [63]. Likewise, Ahn et al. (2003) found that AAV was not associated with any stages of cervical pre-cancer and cancer lesions by in situ hybridization and immunohistochemistry [64]. A case control study in pregnant and non-pregnant women found that AAV infection have any impact on cervical intraepithelial neoplasia development [65]. Moreover, a retrospective case control study by Zheng et al. (2006) found a low proportion of cervical cancer biopsies containing AAV genomes and no evidence that the presence of AAV in cervical smears of healthy women would be associated with a reduced risk of cervical cancer [66]. Finally, an Iranian study from 2017 detected a low proportion of cervical biopsies containing AAV genome (14.8% cervical cancer cases and 14% healthy controls) and no significant difference in correlation between HPV and cervical cancer in the presence or absence of AAV infection was found [67]. A description of the studies assessing the influence of AAV infection in cervical tumorigenesis is shown in Table 1.

**Table 1:** Studies on AAV infection and HPV-related cervical cancer in humans

References	Year	Country	Study population	Samples	Results	Conclusions
[57]	1976	USA	Cases = 120 Control = 128	Serum	Presence of IgG antibodies to AAV type 2–3 complex in genital cancer cases was 14% and in healthy women was 85% (*P* < 0.001).	The percentage of sera with antibodies to AAV was significantly higher in the normal group than in the cancer patients. The role of AAV in possible abrogation of oncogenesis mediated through adenoviruses or herpesviruses is worthy of further investigation.
[58]	1984	Germany	Cases = 83 Control = 50	Serum	The control group exhibited approximately three-fold higher IgG antibodies titers against AAV type 5 than cervical cancer patients.	The data could suggest that AAV infection provides some protection against subsequent cervical cancer development.
[62]	1999	Jamaica USA	Jamaican patients: Cases = 197 (CIN-1 = 105; CIN-3/CA = 92) Control = 94 U.S. patients: Cases = 74 Control = 77	Cervical smear (Jamaica); serum (USA)	None of the 291 cervical specimens from Jamaican subjects tested positive for AAV DNA. No relationship between IgG antibodies to AAV type 2 and presence or grade of neoplasia in either the Jamaican or U.S. cervical cancer cases.	The data provide no evidence that AAV infection plays a role in cervical tumorigenesis or that AAV commonly infects cervical epithelial cells.
[63]	2000	United Kingdom	Cases = 211 (CIN-1 = 83; CIN-3 = 128) Control = 433	Cervical smear	6/433 (1.4%) control cervical smears and 4/211 (1.9%) of CIN (CIN-1 = 2; CIN-3 = 2) contained AAV type 2 DNA. No correlation between AAV and any clinical feature was observed.	AAV DNA is not frequently present in either normal control cervical samples or cervical intraepithelial neoplasia. This does not support the hypothesis that AAV may be protective against cervical cancer.
[59]	2001	USA	Cases = 217 (HSIL = 55; LSIL = 162) Control = 96	Cervical smear	AAV positivity was associated with a significantly reduced risk of HSIL (age and HPV-adjusted odds ratio (aOR) = 0.32) yet not with LSIL (aOR = 0.78); 53.8% of HSIL, 66.9% of LSIL, and 70.7% of controls were AAV+.	AAV may play a protective or inhibitory role in late stage cervical carcinogenesis.
[64]	2003	South Korea	Cases = 92 (CIN-1 = 20; CIN-2 = 24; CIN-3 = 25; invasive = 23) Control (perilesional normal tissues) = 92	Cervical tissue	AAV type 2 was detected in 55% of CIN-1, 84.5% of CIN-2, 52% of CIN-3, and 52.2% of invasive cancer cases. In perilesional normal tissues, AAV was detected in 57.6%, displaying 25% of CIN-1, 83.3% of CIN-2, 52% of CIN-3, and 65.2% of invasive cancer.	The differences in AAV prevalence are not significant between CIN and normal tissues, suggesting no significant correlation between AAV and cervical cancer.
[65]	2004	Croatia	165 nonpregnant and 53 pregnant women with cervical cancer.	Cervical smear	AAV type 2 DNA was found in 6% of the women. AAV infection was more frequently associated with pregnancy (17 versus 2.4%).	There is no evidence that AAV infection has any impact on cervical cancer development. These data point out that HPV could indeed be an AAV helper virus and that AAV as such can be considered sexually transmissible.
[66]	2006	Sweden	Cases = 104 Matched control = 104	Cervical smear; cervical tissue	At baseline, 2% of case-women and 3% of control-women were positive for AAV type 2 DNA. At the time of cancer diagnosis, 12% of case-women and 3% of matched control-women were positive for AAV DNA.	AAV DNA was present in a low proportion of cervical cancers. No evidence that the presence of AAV in cervical smears of healthy women would be associated with reduced risk of cervical cancer.
[60]	2008	Greece	Cases = 93 (CIN-1 = 31; CIN-2/3 = 45; invasive = 17) Control = 280	Cervical smear	AAV infection was confirmed in 16.8% women, and AAV detection was not statistically different between HPV (–) and HPV (+) in the controls. AAV was significantly lower in the HPV (+) relative to the HPV (–) cancer patients.	HPV-infected individuals are less likely to develop cervical neoplasia if AAV is present. AAV probably demonstrates a protective role against the pathogenic consequences of HPV infection.
[61]	2012	Brazil	284 women (HIV positive = 112; HIV negative = 172	Cervical smear	AAV type 2 prevalence was 19.7%, with 18.7% and 20.3% in HIV-positive and -negative women, respectively. AAV was detected with higher frequency in HPV-infected women (*P* < 0.05). The AAV-HPV co-infected women showed a lower rate of atypical squamous cells of undetermined significance or cervical intraepithelial neoplasia development compared with those infected only with HPV.	This is the first report examining AAV in cervical samples of HIV-infected women and indicates that HIV infection does not appear to influence AAV prevalence or AAV-HPV co-infection.
[67]	2017	Iran	Cases = 61 Control = 50	Cervical tissue	AAV type 2 DNA was detected in 7 cases (14%) of healthy controls and 9 specimens (14.8%) of case group.	A low proportion of cervical biopsies from Iranian women contained AAV genome. The correlation between HPV and cervical cancer showed no significant difference in presence or absence of AAV genome in cervix.

CIN-1, low-grade cervical intraepithelial neoplasia; CIN-3/CA, CIN-3/carcinoma in situ or invasive cancer; HSIL, high-grade cervical squamous intraepithelial lesions; LSIL, low-grade cervical squamous intraepithelial lesions

#### AAV infection and reproductive system disorders

Several infections have been linked to miscarriage and other adverse reproductive outcomes. Specifically, 15% of early miscarriages and 66% of late miscarriages have been attributed to infections [68]. In particular, the common occurrence of AAV DNA (and virions) in male and female genital tissues has given rise to the hypothesis that genital AAV infection may be linked to unfavorable impacts on reproduction, including placental complications, spontaneous abortion, and fertility disorders [46, 69–72]. AAV was found fatal for mouse embryos at the early stages of gestation, and transplacental infection was established [73, 74]. In humans, AAV DNA was detected by Tobiasch et al. (1994) in 40% of abortion material during the first trimester of pregnancy but not in the material of abortion from the second or third trimester. They suggested that AAV infection in the uterine mucosa and trophoblast cells may disturb placenta development and promote early miscarriage [46]. In agreement with previous results, Malhomme et al. (1997) showed the presence of AAV DNA in 69% of materials from early abortions [75]. Koi et al. (2001) hypothesized that viral infection of extravillous trophoblast cells might hinder placental penetration of the uterine wall, resulting in situations such as spontaneous miscarriage, pre-eclampsia, and premature birth [76]. In a case-control study by Arechavaleta-Velasco et al. (2006), AAV DNA was found more frequently in trophoblast cells from cases of severe pre-eclampsia (55%) than from normal term deliveries (19%, *P* = 0.002) [70]. Two years later, they demonstrated that primary or reactivated AAV infection (maternal IgM seropositivity) early in pregnancy was associated with adverse reproductive outcomes linked to placental dysfunction, including pre-eclampsia, stillbirth, and spontaneous preterm delivery [69]. Kiehl et al. (2002) demonstrated AAV infection in embryo-derived tissue from Brazilian patients and further suggested a role of AAV in miscarriage and trophoblastic disease [71]. Also, in Brazil, Pereira et al. (2010) detected AAV DNA in 28.6% and 2.4% (*P* < 0.05) of the spontaneous and intentional abortions, respectively, suggesting an association between AAV and spontaneous miscarriage [72]. In contrast to these findings, Friedman-Einat et al. (1997) and Matovina et al. (2004) did not detect AAV DNA in any case of spontaneous abortion [77, 78]. Consistent with these studies, Sayyadi-Dehno et al. (2019), by analyzing the presence of AAV DNA in 81 therapeutic and 83 spontaneous abortions, found no statistically significant difference between the two groups [45].

Concerning to fertility factors, Rohde et al. demonstrated for the first time in 1999 the occurrence of AAV infection in human semen and suggested that sperm motility may be affected by the presence of AAV [79]. In 2001, Erles e*t al*. showed an increased incidence of AAV infection with abnormal semen analysis, suggesting a role for AAV infection in male infertility, possibly interfering with spermatozoa development [80]. Furthermore, Mehrle et al. (2004) demonstrated that AAV DNA is integrated into testis tissue samples [81]. In contrast, Schlehofer et al. (2012) investigated AAV DNA in semen samples and endocervical material of 280 individuals of subfertile couples (146 males and 134 females), and no associations between AAV and other infectious pathogens, semen quality or subsequent fertility issues were indicated [82]. The studies investigating the association between AAV infection and adverse reproductive outcomes in humans are described in Table 2.

**Table 2:** Studies on AAV infection and reproductive system disorders in humans

References	Year	Country	Study population	Samples	Results	Conclusions
[46]	1994	France	108 sera (24 women presenting with early miscarriage; 23 women with lesions of the cervix uteri; 61 controls). 30 biopsies of the cervix uteri (10 normal tissue; 5 lesions of the endometrium; 15 cervical cancer). 30 curettage material of spontaneous abortion.	Serum; biopsy of uterus; tissue material from spontaneous abortion	AAV type 2 DNA was amplified in histological sections of 19 of 30 biopsies of the uterine mucosa. AAV DNA was detected in abortion material during the first trimester of pregnancy (12/30 cases were positive) but not in material of abortion from the second or third trimester (9 cases). The prevalence of IgM antibodies to AAV type 2 was elevated in cases of spontaneous abortion (first trimester, 29.1%), and in women with cervical cancer (30.4%) compared to the control group (9.9%).	In view of the presence of AAV DNA and proteins in placenta tissue, serological tests might be useful to assess further the hypothesis of a possible role of AAV infection in spontaneous abortion.
[75]	1997	France	13 histological sections of the cervix uteri; 9 endometrium biopsies; 26 samples of abortion material; 2 samples of curettage material of extrauterine gravidity; 1 sample of socially indicated abortion.	Cervical tissue; abortion material	HPV DNA was detected in approximately 60% of paraffin sections from uterus biopsies and cervical lesions containing AAV type 2 DNA and in approximately 70% of material from early miscarriage.	HPV may be a helper virus for AAV.
[77]	1997	Israel	15 nasopharyngeal aspirates from symptomatic patients; 7 swab or fluid specimens from vesicles of patients suspected of having varicella-zoster virus infections; 21 human papilloma virus-positive genital biopsy specimens; 61 genital swab specimens from women suspected of having herpes simplex virus; 62 samples of first trimester aborted material (38 spontaneous and 24 induced abortions); 11 samples of chorionic villi; 3 lots of cultured human embryonic cells.	Different clinical samples.	AAV type 2 sequences were detected only in samples (n = 11) taken from the genital tracts of women suspected of having herpes infection and not in any of the other types of samples.	Our study demonstrates the presence of AAV in the female genital tract. However, in contrast to a previous report, we did not find solid evidence of its replication in maternal or embryonal tissues from the first trimester of pregnancy. The questions of a potential pathogenic etiology of AAV and the interaction with HSV remain open.
[79]	1999	Germany	Men with diagnosed infertility = 30 Control = 8	Semen	AAV DNA was detected in 30% (9/30) of the ejaculates from the infertile men. No AAV DNA was found in the ejaculates from the 8 control subjects. In 8 of 9 samples, AAV DNA could be found only in the spermatozoal fraction of the specimen. Seven of 9 semen specimens that contained viral DNA also demonstrated oligoasthenozoospermia. Both AAV and HPV DNA was found in the spermatozoal fraction of 3 of 30 specimens.	The data demonstrate for the first time the occurrence of AAV infection in human semen. Sperm motility seems to be affected by the presence of AAV.
[80]	2001	Germany	95 men (with history of infertility = 73; without history of infertility = 22) 17 men (with malignant melanoma = 3; benign tumor = 5; stone = 1; adenoma of the prostate = 1; no diagnosis = 7). 38 azoospermic men. 57 female partners.	Semen; cervical smear; urethral smear; testicular biopsy	AAV DNA was detected in 38% (28/73) of ejaculates from men with abnormal semen analyses and in 4.6% of normal semen samples (1/22, *P* > 0.003). In testes, AAV DNA was detected in 10 out of 38 biopsies from infertile men (26%), and in 2 out of 8 orchidectomy samples.	The data show an increased incidence of AAV infection with abnormal semen analysis. Detection of AAV DNA in the testes might point to a role for AAV infection in male infertility, possibly by interfering with spermatozoa development.
[71]	2002	Brazil	78 paraffin-embedded tissue samples, including histologically confirmed hydatiform moles (42 complete, 4 partial, 3 invasive, 14 choriocarcinomas, and 15 materials from spontaneous abortion.	Tissue of hydatiform moles, choriocarcinomas, and spontaneous abortion.	AAV DNA was found in 43 samples (28/49 hydatiform moles, 4/14 choriocarcinomas, 11/15 miscarriage material).	These findings confirm AAV infection of embryo-derived tissue in humans and further suggest a role of AAV in miscarriage and trophoblastic disease.
[78]	2004	Croatia	108 women admitted to the hospital for threatened miscarriage.	Placental tissue	No detection of AAV DNA.	No influence of AAV infection in miscarriages during the first trimester of pregnancy.
[81]	2004	Germany	2 patients with prostate cancer.	Testis tissue	AAV DNA is present in an integrated form in testis tissue. A detailed analysis revealed integration within sequences of the so-called AAVS1 region on chromosome 19.	AAV DNA can integrate also after natural infection, and that integration occurs within the AAVS1 region, at least in some cases.
[70]	2006	USA	Cases of preeclampsia = 40 Control = 27	Histological sections from the basal plate region of placentas, and trophoblast cells.	AAV type 2 DNA was found more frequently in trophoblast cells from cases of severe preeclampsia (22/40) than from normal term deliveries (5/27, *P* = 0.002).	AAV infection is a previously unidentified cause of placental dysfunction.
[69]	2008	USA	Cases = 78 (34 spontaneous abortions; 24 spontaneous preterm deliveries; 20 women with at least one outcome usually attributed to placental dysfunction) Control = 106	Serum	First trimester maternal IgM seropositivity to AAV type 2 was 5.6 times more prevalent among pre-eclampsia/IUGR/stillbirth cases (*P* = 0.0004) and 7.6 times more prevalent among preterm deliveries (*P* < 0.0001) than among controls. AAV infection (IgG seropositivity) was not associated with adverse pregnancy outcomes.	Primary or reactivated AAV infection (maternal IgM seropositivity) early in pregnancy was associated with adverse reproductive outcomes associated with placental dysfunction, including pre-eclampsia, stillbirth, and spontaneous preterm delivery.
[72]	2010	Brazil	Spontaneous abortion group = 68 Intentional abortion group = 13	Decidual and chorionic tissues.	AAV type 2 was detected in 28.4% (23/81) of the abortion cases for at least one of the decidual or ovular fragments, 32.3% (22/68) of the spontaneous and 7.7% (1/13) of intentional abortions.	The presence of AAV in decidual or trophoblastic cells in cases of abortion, as observed by i*n situ* hybridization, implies that the virus could jeopardize the pregnancy. The significant predominance in spontaneous cases suggests possibly a causal association between AAV and abortion.
[82]	2012	Germany	146 male and 134 female partners of asymptomatic subfertile couples.	Semen samples and endocervical material	AAV DNA was detected in 20 out of 134 (14.9%) cervical swabs and in 29 out of 146 (19.9%) semen samples. 3.8% (5/133) couples were AAV DNA positive in both semen and endocervical materials. The presence of AAV DNA in semen was not significantly related to semen quality, nor was it coupled to the presence of AAV in the endocervical material of female partners. AAV DNA in endocervical material was not related to a reduced quality of cervical mucus or to other female infertility factors.	The presence of AAV DNA in semen samples or endocervical swabs showed no significant association with clinically relevant infertility factors.
[45]	2019	Iran	Therapeutic abortions = 81 Spontaneous abortions = 83	Placental tissues	62 (38.2%) of 164 abortions were AAV positive, including 35 (21.6%) spontaneous abortions and 27 (16.6%) therapeutic abortions.	There was no statistically significant difference between the presence of the AAV genome in spontaneous and therapeutic abortions.

IUGR, intrauterine growth restriction

#### AAV infection and hepatocellular carcinoma

Liver cancer is expected to be the sixth most significant cause of cancer and the third leading cause of cancer-related mortality globally in 2020, with a reported 905,677 new cases and 830,180 deaths, posing a severe global health burden [83]. Hepatocellular carcinoma (HCC) represents 90% of all primary liver tumors [84]. HCC most commonly occurs due to chronic liver inflammation leading to liver cirrhosis, mainly caused by viral hepatitis, alcohol misuse, and nonalcoholic fatty liver disease [85]. The most common risk factor for developing HCC, accounting for around 50% of cases, is chronic infection with the hepatitis B virus (HBV) [86]. The evolution of a liver disease in HBV-infected patients (as well as for hepatitis C) is explained by the constant and unsuccessful attempts of the immune system to clear the virus, resulting in chronic liver damage [87]. However, apart from this common indirect mechanism, the development of 10 to 30% of HBV-associated HCC in a liver without cirrhosis suggests that HBV may also have direct oncogenic properties [88]. One of the genomic features of HBV-related HCC is the presence of frequent viral integrations in genes involved in carcinogenesis such as *TERT*, *MLL4*, and *CCNE1* [88].

It has been demonstrated that the liver is the main site of infection of AAV [47]. Interestingly, in small sets of patients with HCC, AAV showed insertional oncogenic mutagenesis similar to HBV, with a common hot spot of viral insertion within *TERT*, *CCNE1*, and *CCNA2* cancer driver genes, leading to their overexpression [44, 89, 90]. Two different mechanisms explained the oncogenic effects of AAV clonal integrations. First, the integrated AAV sequence typically comprises transcription factor binding sites as well as viral enhancers [91], which causes an oncogene to be strongly overexpressed nearby (*CCNE1* or *TERT*) [44, 92]. Second, due to the usage of an alternate transcription start site (TSS) or the viral poly-A site, integrated viral sequences may cause the synthesis of truncated transcripts (for integrations in *CCNA2* and *TNFSF10*, respectively) [93]. Remarkably, a region common to all inserted AAV sequences has recently been identified as a liver-specific enhancer-promoter element [91]. Although this region is missing in the majority of rAAV vectors currently used for gene therapy, animal studies have shown that integrated rAAV vectors can cause clonal growth and play a role in carcinogenesis [94–96]. On the other hand, no evidence of malignancy was observed from hundreds of normal mice treated with rAAV vectors [97, 98]. Likewise, big animals treated with relatively high doses of rAAV vectors, such as dogs and nonhuman primates, showed no malignancy [99, 100].

In La Bella et al. (2019), the analysis of tumor and non-tumor liver tissues of 1,461 patients revealed the presence of AAV DNA in 21% of patients, mainly in young women without cirrhosis [89]. AAV was found in only 8% of the examined tumors; the proportion was the same in malignant and benign tumors. However, the malignant tumors had more copies per cell due to clonal AAV integrations. In 2% of the HCC cohort, 19 AAV clonal integrations were found, and hardly any episomal virus was detected. All of these HCCs originated on non-cirrhotic livers with no known other etiologies. This study demonstrated that, although rare, AAV integration in specific regions of the human genome can promote hepatocarcinogenesis in a non-cirrhotic liver, confirming that AAV is associated with HCC occurrence without other etiologies via an insertional mutagenesis mechanism [89]. Additionally, Park et al. (2016) found AAV DNA in only 0.7% (n = 2/289) of Korean patients with HCC. Although the two patients showed no signs of liver cirrhosis, both presented other HCC risk factors, such as chronic HBV infection. The possibility of an interaction between HBV and AAV on hepatocarcinogenesis was suggested [101]. Tatsuno et al. (2019) performed virome capture sequencing using HCC and liver samples obtained from patients with prior HBV or chronic HBV infection to investigate the integration of HBV and AAV into the human genome as a possible oncogenic event. In this study, *CCNE1* and *CCNA2* were transcriptionally activated by AAV in prior HBV infection, suggesting that despite the seroclearance of HBV surface antigen, such patients are at risk of developing HCC [90]. Table 3 summarizes the studies demonstrating the association between AAV infection and the development of HCC in humans.

**Table 3:** Studies on AAV infection and HCC development in humans

References	Year	Country	Study population	Samples	Results	Conclusions
[44]	2015	France	HCC samples and corresponding non-tumor tissues = 193	Liver tissue	Clonal integration of AAV type 2 was found in 11 of 193 HCCs. Integrations occurred in the known cancer driver genes *CCNA2*, (four cases), *TERT* (one case), *CCNE1* (three cases), *TNFSF10* (two cases) and *KMT2B* (one case), leading to overexpression of the target genes. Tumors with AAV integration mainly developed in non-cirrhotic liver (9 of 11 cases) and without known risk factors (6 of 11 cases), suggesting a pathogenic role for AAV in these patients.	AAV is a DNA virus associated with oncogenic insertional mutagenesis in human HCC.
[101]	2016	South Korea	HCC patients = 289 (159 hepatitis-B-related cases; 16 hepatitis-C-related cases; 114 viral serology-negative cases)	HCC tissue	AAV type 2 DNA was detected in 0.7% (2/289) of the patients. AAV-related HCC showed no signs of liver cirrhosis.	AAV-associated HCC was very rare in Korean patients with HCC. This study is the first to report the clinical characteristics of Korean patients with AAV-associated HCC. These findings suggest epidemiologic differences in viral hepatocarcinogenesis between Korean and European patients.
[90]	2019	Japan	HCC patients = 243 (73 prior HBV without HCV infection; 81 chronic HBV; 56 prior HBV with HCV infection; 33 non-B non-C cases as negative controls)	Liver tissue	AAV type 2 was found to integrate into 3 genes in two prior HBV and 1 non-B non-C patients. *CCNE1* and *CCNA2* were targeted by AAV2 only in prior HBV, while *SLC6A5* was integrated in a non-B non-C patient.	Despite the seroclearance of hepatitis B surface antigen, HBV or AAV integration in prior HBV was not rare; therefore, such patients are at risk of developing HCC.
[89]	2020	France	Patients with liver tumor = 1461 (936 HCC; 225 hepatocellular adenomas; 97 focal nodular hyperplasia; 87 hepatoblastoma or transitional tumors; 46 cholangiocarcinoma; 36 fibrolamellar carcinoma; 34 other tumors)	Liver tissue	AAV types 2 and hybrid 2/13 DNA was detected in 21% of the patients, including 8% of the tumor tissues. Episomal viral forms were found in 4% of the non-tumor tissues, frequently associated with viral RNA expression and HHV-6. In 30 HCC, clonal AAV insertions were recurrently identified in *CCNA2*, *CCNE1*, *TERT*, *TNFSF10*, *KMT2B* and *GLI1/INHBE*. AAV insertion triggered oncogenic overexpression through multiple mechanisms that differ according to the localization of the integration site.	Clonal AAV insertions were positive selected during HCC development on noncirrhotic liver challenging the notion of AAV as a nonpathogenic virus.

## Conclusion

AAV-based gene therapy has significantly benefited from the fundamental knowledge developed in AAV research over the past 30 years. This progress has led to the development of several rAAV vectors, that are either undergoing clinical trials or have already received FDA approval, including Glybera, Luxturna, and Zolgensma. However, given that 35–80% of the world’s population is seropositive for neutralizing antibodies against one or more forms of AAV, it is remarkable that many issues concerning the AAV life cycle in vivo remain unanswered. Due to the intricate nature of the cause-and-effect link, it is challenging to make any firm inferences about the impact of AAV infection on human health. Although AAV is considered non-pathogenic, several reports describe AAV infection in association with adverse reproductive outcomes. The AAV link with tumor development is controversial. Some studies report an oncogenic effect of AAV infection (as in hepatocellular carcinoma), and others suggest a tumor suppressive role (as in HPV-related cervical cancer). Of note, natural infections with wild-type AAV have no demonstrable connection with the administration of current rAAV vectors since a significant portion of the native AAV genome is deleted during the construction of AAV-based vectors for gene therapy. In addition, no adverse event, including cancer of any kind, has ever been documented in clinical studies performed thus far using rAAV vectors [105,106]. Nevertheless, considering the extensive usage of rAAV vectors in liver-targeted gene therapy and its potential for insertion into the human genome, patients treated with rAAV vectors should be followed longitudinally to monitor long-term consequences and determine the risk of HCC development.

## Data Availability

Not applicable.
